# Haplotype-resolved chromosome-scale genomes of the Asian and African Savannah Elephants

**DOI:** 10.1038/s41597-023-02729-4

**Published:** 2024-01-11

**Authors:** Minhui Shi, Fei Chen, Sunil Kumar Sahu, Qing Wang, Shangchen Yang, Zhihong Wang, Jin Chen, Huan Liu, Zhijun Hou, Sheng-Guo Fang, Tianming Lan

**Affiliations:** 1 BGI Life Science Joint Research Center, Northeast Forestry University, Harbin, 150040 China; 2 State Key Laboratory of Agricultural Genomics, Key Laboratory of Genomics, Ministry of Agriculture, BGI Research, Shenzhen, 518083 China; 3 College of Life Sciences, University of Chinese Academy of Sciences, Beijing, 100049 China; 4 Southwest Survey and Planning Institute of National Forestry and Grassland Administration, Kunming, 650031 China; 5 Asian Elephant Research Center of National Forestry and Grassland Administration, Kunming, 650031 China; 6 MOE Key Laboratory of Biosystems Homeostasis & Protection, State Conservation Centre for Gene Resources of Endangered Wildlife, College of Life Sciences, Zhejiang University, Hangzhou, 310058 China; 7 Guangdong Provincial Key Laboratory of Genome Read and Write, BGI Research, Shenzhen, 518083 China; 8 China National GeneBank, BGI Research, Shenzhen, 518083 China; 9 College of Wildlife and Protected Area, Northeast Forestry University, Harbin, 150040 China

**Keywords:** Comparative genomics, Conservation biology

## Abstract

The Proboscidea, which includes modern elephants, were once the largest terrestrial animals among extant species. They suffered mass extinction during the Ice Age. As a unique branch on the evolutionary tree, the Proboscidea are of great significance for the study of living animals. In this study, we generate chromosome-scale and haplotype-resolved genome assemblies for two extant Proboscidea species (Asian Elephant, *Elephas maximus* and African Savannah Elephant, *Loxodonta africana*) using Pacbio, Hi-C, and DNBSEQ technologies. The assembled genome sizes of the Asian and African Savannah Elephant are 3.38 Gb and 3.31 Gb, with scaffold N50 values of 130 Mb and 122 Mb, respectively. Using Hi-C technology ~97% of the scaffolds are anchored to 29 pseudochromosomes. Additionally, we identify ~9 Mb Y-linked sequences for each species. The high-quality genome assemblies in this study provide a valuable resource for future research on ecology, evolution, biology and conservation of Proboscidea species.

## Background & Summary

In recent decades, there has been a growing interest in the body size of proboscideans, as it is closely associated with a variety of biological functions due to its high correlation with mass^1^. Currently, there are two families within Proboscidea, comprising three species: the Asian elephant, the African savannah elephant, and the African forest elephant (*Loxodonta cyclotis*). The population of proboscis animals has been rapidly decreasing due to factors like poaching and hunting. As a result, they are now classified as critically endangered and endangered on the IUCN red list (https://www.iucnredlist.org/). People’s preference for ivory has also caused some unique evolutionary changes in proboscis animals, such as a substantial increase in the proportion of female African elephants without tusks and a gradual decrease in the size of tusks in male African elephants^2^. In addition, the swift expansion of economic crop cultivation areas has led to habitat fragmentation, emerging as a significant peril to wild populations^3^. A growing quantity of elephants are coming out of the forest and regularly exploring villages and residential areas. An increasing number of elephants are coming out of the forest and frequently venturing into villages and residential areas. As a result, there have been occasional occurrences of crop damage, as well as harm to humans and animals. The escalating human-elephant conflict poses a significant challenge to conservation efforts and is detrimental to the healthy development of the elephant population. Additionally, variations in the population of large mammals exert a greater impact on other animals within their habitat. Therefore, the protection and conservation of elephants has become a focus of ecological diversity efforts. In the era of transitioning from conservation genetics to conservation genomics^4–7^, high-quality reference genome is of vital importance to improve the evaluation of the full spectrum of genomic diversity, inbreeding and outbreeding depression, local adaptation and genetic loads^8–11^. Furthermore, this genome assembly will provide a valuable resource for studying the ecology and evolution of specific species^12,13^.

Rapid advances in high-throughput sequencing technologies over the past decade have opened new avenues for addressing the genetic basis of natural population adaptation and speciation^14^. The use of genetic data has proven valuable in delineating taxa that cannot be identified based on morphology alone^15–17^. In the case of endangered animals, the analysis of haplotype can assist in detecting hidden signals of inbreeding depression, providing crucial insights for conservation initiatives^18^. Therefore, obtaining high-quality elephant genomes will be important for elucidating the genetic mechanisms underlying the species’ distinct biological characteristics and complexity, as well as for informing conservation strategies aimed at preserving these species. Although the draft genomes of the two elephants have been released before^19,20^, the recent HiFi sequencing technology greatly improves the genome quality and supplies haplotype-resolved reference genome^20–22^.

In this study, we generated two chromosome-level and haplotype-resolved genome assemblies of the Asian Elephant and African Savannah Elephant using PacBio HiFi long-reads, DNBSEQ short-reads, and Hi-C sequencing data. The assembled genome sizes were 3.38 Gb and 3.31 Gb for the Asian elephant and African savanna elephant, with the N50 length of 130 Mb and 122 Mb, respectively. These results are significantly improved compared to the published genomes^14,15^. Approximately 97% of the assembled sequences were anchored to 29 pseudochromosomes. The collinearity analysis of the chromosome-level genomes of the two species is consistent with the results of published karyotype studies^23^, which verifies the accuracy of genome assembly in this study. Using a combination of *de novo* prediction, homology-based search, and transcriptome-assisted method, we annotated 22,177 and 22,142 protein-coding genes in genomes of the Asian elephant and African savanna elephant, respectively. Additionally, we identified ~ 9 Mb of Y-linked sequences from both of the two elephant genomes by combining the sex-determining region (*SRY*) and chromosomal synteny evidence. the two high-quality elephant reference genomes produced in this study are a valuable resource for future research on the ecology, evolution, biology, and conservation of Proboscidea species. The two high-quality elephant reference genomes in this study are a valuable resource for future research on ecology, evolution, biology and conservation for Proboscidea species. The genomes hold the potential to delve into a diverse array of subjects, offering an opportunity to enhance our comprehension of these incredible creatures and bolster efforts for their conservation.

## Methods

### Sample collection and ethics statement

Blood samples from *E. maximus* and tissue samples from *L. africana* were provided by the Asian Elephant Research Center of National Forestry and Grassland Administration of China and Harbin North Forest Zoo, Heilongjiang Province, China. A portion of the fresh sample (blood sample from an Asian elephant, and muscle tissue sample from an African savannah elephant) was taken out and treated with formaldehyde for the cross-linking of the chromatin, and then stored at −80 °C for Hi-C sequencing. The remaining sample was immediately frozen in liquid nitrogen for 30 min and then transferred to the −80 °C refrigerator for PacBio sequencing, DNBSEQ sequencing and RNA-seq sequencing. Sample collection, follow-up experiments and research design in this study were all approved by the Institutional Review Board of BGI (BGI-IRB E22017).

### Nucleic acid extraction, library construction and sequencing

Total genomic DNA was extracted using a Dneasy Blood and Tissue Kit (Qiagen, USA) for whole genome sequence (WGS) library. Total RNA from blood and muscle tissue were extracted using Trlzol reagent (Invitrogen, USA), and cDNA libraries were reverse-transcribed using 200–400 bp RNA fragments (Supplementary table 1). The concentration of nucleic acid was detected by Qubit 2.0 Fluorometer (Life Technologies, USA), and RNA integrity was evaluated using an Agilent 2100 Bioanalyzer System (Agilent, USA). These two types of libraries were subjected to paired-end sequencing using a DNBSEQ-T1 sequencer (MGI tech, Shenzhen, Guangdong, China). A 15k library was constructed by using high-quality DNA samples (main band > 30 kb) and sequenced with a Pacbio Sequel II platform (Novogene, Tianjin, China). Low-quality reads and sequencing-adaptor-contaminated reads were removed. Finally, a total of ~100 GB clean data were used to assemble the two genomes (Table 1). Cross-linked samples were prepared with dnpII restriction endonuclease for Hi-C library and PE-sequenced by Illumina Hiseq.

**Table 1:** Sequencing stats.

Species	Library types	Insert size (bp)	Data size (Gb)	Read length (bp)	Depth (×)
***E***. ***maximus***	PacBio	15000~18000	103.74	17175	30.71
	WGS	300~500	386.20	100	114.34
	Hi-C	/	200.28	150	59.32
	RNA-seq	250~300	6.28	150	/
***L***. ***africana***	PacBio	15000~18000	107.41	16880	32.41
	WGS	300~500	281.76	100	85.02
	Hi-C	/	200.66	150	60.55
	RNA-seq	250~300	5.88	150	/

### Genome assembly

**Fig. 1:**
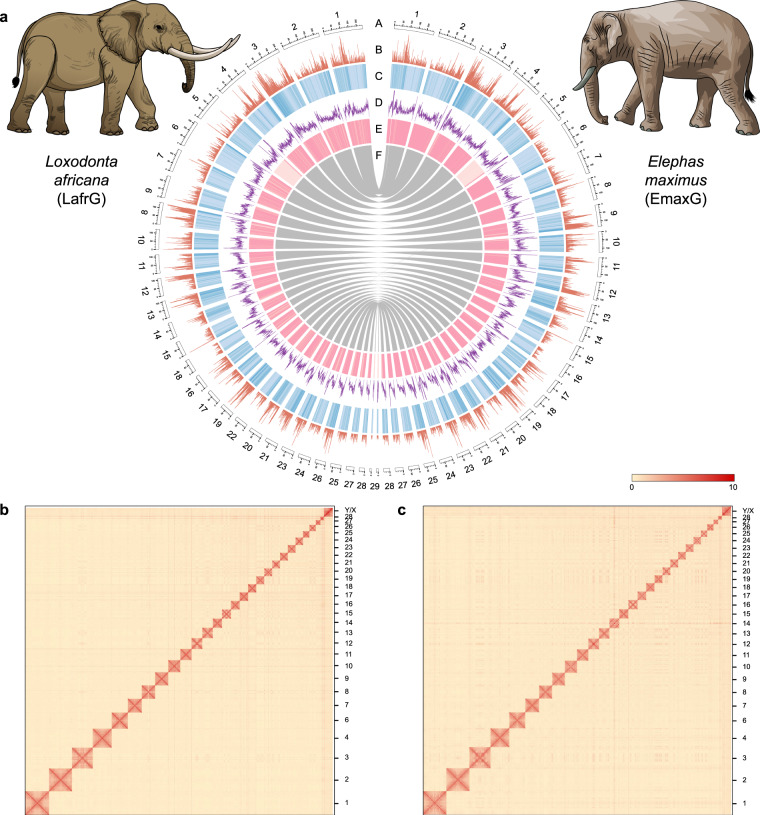
Characteristics of the chromosome-scale genomes of the Asian (*Elephas maximus*) and African Savannah Elephant (*Loxodonta africana*). (**a**) Circos plot of genome assembly. A) Pseudo-chromosomes; B) gene density; C) GC content; D) repeat number; E) sequencing depth (~100 Gb DNBSEQ reads aligned to the genome); F) chromosome synteny (keep the longest 25,000). (**b**) Hi-C intra-chromosomal contact map of the *L. africana* haploid genome assembly. (**c**) Hi-C intra-chromosomal contact map of the *E. maximus* haploid genome assembly. Hi-C interactions within and among chromosomes were drawn based on the chromatin interaction frequencies between pairs of genomic regions.

To estimate the genome size, a total of ~100 Gb DNBSEQ short reads were used for analysis by kmerfreq (v5.0)^24^. The final estimated genome size is 3.44 Gb for *E. maximus* and 3.50 Gb for *L. africana* (Supplementary Fig. 1). The heterozygous and haplotype draft genomes of the two elephants were assembled by using Hi-C and PacBio sequencing data in hifiasm (v0.16.1)^25^. In the genome polishing stage, minimap2 (v2.17)^26^ and NextPolish (v1.4.0)^27^ were mainly used to improve the accuracy of single bases by three rounds of HiFi reads and two rounds of DNBSEQ reads. Redundancy removal of genomes was performed by Purge_dups (v1.2.5)^28^. The burrows-Wheeler Aligner (BWA, v0.7.17) *mem* algorithm^29^ was used for Hi-C sequencing reads mapping to the primary genome. The Juicer (v1.5)^30^ was used for Hi-C data quality control, and the 3d-DNA pipeline (v190716)^31^ was finally used to concatenate and review the scaffolds to the chromosome-scale genome. Finally, two hybrid genomes composed of 29 pseudo-chromosomes and two sets of haplotigs composed of 28 pseudo-chromosomes were obtained, and the average Hi-C mounting rate reached 97.28 ± 0.60% (Fig. 1, Supplementary Tables 3, 4). Basic assembly statistics, reaching 130 Mb and 122 Mb for Scaffold N50, show a significant improvement over published Elephant genomes (Table 2, Supplementary table 4)^14,15^.

**Table 2:** Comparison of the assembly statistics among the genomes assembled in this study (EmaxG and LafrG) and the previously published elephant genomes^19,20^.

Assembly Level	Parameters	ASM1433276v1	mEleMax1	EmaxG	Loxafr3.0	mLoxAfr1	LafrG
Scaffold	Maximal length (bp)	14,655,169	243,826,021	**244,444,223**	129,759,341	241,857,137	**238,728,000**
	N90 (bp)	598,295	79,875,053	**79,328,989**	6,641,774	50,538,043	**76,018,500**
	N50 (bp)	2,767,252	127,432,672	**130,469,234**	46,401,353	119,600,562	**122,446,792**
	number > = 100 bp	6,948	64	**45**	2,352	880	**743**
	number > = 2 kb	4,730	63	**45**	2,352	880	**695**
	Ratio of Ns	0.05672	0.00098	**0.00002**	0.02446	0.00104	**0.00009**
	Genome size (bp)	3,128,770,357	3,401,247,148	**3,377,773,971**	3,196,738,102	3,540,893,228	**3,314,059,562**
Contig	Maximal length (bp)	399,444	208,165,719	**236,900,082**	567,621	229,222,375	**232,641,661**
	N90 (bp)	1,096,871	17,331,029	**14,785,507**	18,508	4,903,835	**13,880,000**
	N50 (bp)	192,368	87,987,108	**77,194,844**	69,023	82,653,632	**71,750,044**
	number > = 100 bp	2,123,890	190	**176**	95,866	1,111	**1,324**
	number > = 2 kb	342,262	189	**176**	85,812	1,111	**1,267**
	Genome size (bp)	2,951,305,338	3,397,913,467	**3,377,713,718**	3,118,542,609	3,537,204,660	**3,313,776,217**

By identifying the sex-determining region of Y-chromosome (*SRY*) and examining the chromosomal synteny between species using (MUMmer, v4.0.0rc1)^32^, we also discovered two Y-linked regions of ~9 Mb each, which were verified on the DNBSEQ reads depth distribution (Supplementary Fig. 2).

### Repeat regions prediction

Transposable elements (TEs) and other repetitive elements were identified using a combination of homology-based and *de novo* approaches. For the homology-based approach at both the DNA and protein levels, the genome assembly was aligned to the known repeat database REPBASE (v21.01) using RepeatMasker^33^ (v4.0.5), RepeatProteinMask^33^ and Tandem Repeats Finder (TRF)^34^ (v4.07b). For the *de novo*-based approach, RepeatModeler^35^ (v2.0) and LTR_retriever^34^ were used to construct a *de novo* repeat library. We found that the Asian elephant and African savanna elephant genomes contained 69.16% and 70.32% TEs, respectively, with the proportions of each type being similar across these two species (Table 3, Supplementary Tables 5, 6). Long Interspersed Nuclear Elements (LINEs) accounted for most TEs, occupying about ~54% of the genome. All repetitive elements were masked for gene annotation.

**Table 3:** Statistics of the repeat elements.

Type	EmaxG		LafrG	
	Length (Bp)	% in genome	Length (Bp)	% in genome
DNA	98,864,930	2.93	62,963,140	1.90
LINE	1,818,056,280	53.82	1,806,545,100	54.51
SINE	220,195,495	6.52	147,446,993	4.45
LTR	502,367,633	14.87	735,879,514	22.20
Other	119	0.00	115	0.00
Unknown	37,348,324	1.11	21,973,580	0.66
Total	2,375,283,282	70.32	2,291,962,134	69.16

### Annotation of protein-coding genes

We combined homology-based, *de novo* and transcriptome-based methods to predict assembled gene content. In a homology-based approach, GeneWise^36^ (v2.4.1) was used to map 14 closely related or high-quality protein sequences, including *Homo sapiens, Mus musculus, Suncus etruscus, Equus caballus, Felis catus, Phyllostomus discolor, Sus scrofa, Choloepus didactylus, Dasypus novemcinctus, Trichechus manatus latirostris, Orycteropus afer afer, Elephantulus edwardii, Echinops telfairi*, and *Chrysochloris asiatica*, available in the NCBI database, to two assembled genomes with an E-value cutoff of 1e-5. In the *de novo* method, we run the repeat-masked genome using Augustus^37^ (v3.0.3). In the transcriptome-based method, transcripts were assembled using StringTie^38^ (v1.3.3b) based on clean RNA-seq data. The final protein-coding gene set was generated using the MAKER pipeline^39^ (v3.01.03) by combining high-quality homology-based, *de novo* and RNA-seq supported genes. Based on the above methods, 22177 genes were annotated in the Asian elephant genome, while 22142 genes were annotated in African elephant genome (Table 4).

**Table 4:** Protein-coding gene statistics.

Type	EmaxG	LafrG
The total number of gene	22,177	22,142
The average of mRNA length	41,616.04	40,311.95
The average of cds length	1,570.95	1,553.39
qThe total number of exon	192,463	190,989
The average of exon number	8.68	8.63
The average of exon length	181.02	180.09
The total number of intron	170,286	168,847
the average of intron number	7.68	7.63
The total intron length	888,079,848	858,191,881
The average of intron length	5,215.23	5,082.66

### Annotation of gene function

**Fig. 2:**
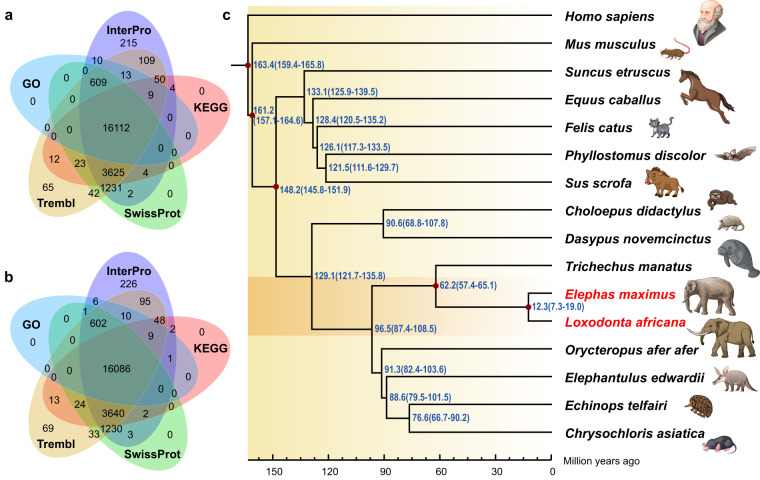
Genome Annotation Statistics. (**a**) Venn diagram of *E. maximus* gene counts with homology or functional classification by each method. (**b**) Venn diagram of *L. africana* gene counts with homology or functional classification by each method. (**c**) A phylogenetic tree based on single-copy genes from 16 species showing the estimated divergence time (Silhouette from https://www.freevectors.net/free-vectors/animals).

Functional annotations of protein-coding genes were carried out using BLAST (e-value cut-off of 1e-5) against publicly available databases, including the Swiss-Prot, TrEMBL, Gene ontology (GO) terms and KEGG database. InterProScan^40^ (v5.52–86.0) was used to predict domains and motifs. 99.81% of the genes in the gene sets of both elephant species were fully annotated in the five above-mentioned databases (Fig. 2a,b, Supplementary Table 7). In addition, noncoding RNA (ncRNA) genes, including miRNA, tRNA, snRNA and rRNA, were predicted in the assembled genome. tRNA genes were identified using tRNAscan-SE^41^ (v1.3.1). snRNA and miRNA genes were detected by searching the reference genome sequences against the content of the Rfam database (Release 12.0) using BLAST (Supplementary Table 8).

### Phylogenetic comparative analysis

We performed a comparative genomic analysis between the *E. maximus*, *L. africana* and 14 reference species used in the previous step, among which *Homo sapiens* was set as an outgroup. First, the longest transcript of each gene from each species was used to perform all-to-all BLAST^42^ (v2.2.26) analysis with the parameter “-p blastp -m8 -e 1e-5 -F F”. Then, genes were clustered using Treefam^43^ (v1.4) pipeline with hierarchical clustering on a sparse graph. Finally, 2365 single-copy genes were identified (Supplementary Fig. 3). These single-copy genes were used to construct a Maximum-Likelihood (ML) phylogenetic tree using IQTREE^44^ (v1.6.12), with the best-fit evolutionary substitution model (GTR + F + R4) using ModelFinder^45^. To estimate the divergence time between C. versicolor and the other 14 species, we used MCMC Tree^46^ (v4.5) implemented in the PAML package. Sequences for 2365 single-copy genes were used as the input file for MCMC Tree, and multiple fossil times were u from Timetree (http://www.timetree.org/). The Markov chain Monte Carlo (MCMC) process was run for 1,500,000 iterations of 150 after a burn-in of 500,000 iterations with a sampling frequency (Fig. 2c).

## Data Records

The chromosome-scale genome sequences of two elephant species are available at the NCBI GenBank under the accession number GCA_033060105.1^47^ (EmaxG) and GCA_033060095.1^48^ (LafrG), and the haplotype-resolved genome sequences are also available at NCBI (EmaxH1: GCA_032718755.1^49^, EmaxH2: GCA_032718585.1^50^, LafrH1: GCA_032717405.1^51^, LafrH2: GCA_032717415.1^52^). The annotation files generated in the current study are available in the figshare database^53^. The raw data that support the findings in this study have been deposited into National Genomics Data Center (NGDC)^54^ Genome Sequence Archive (GSA)^55^ database with the accession number CRA012221^56^ under the BioProject accession number PRJCA018778. All the above sequencing and analysis data in this study is also available in CNGB Sequence Archive (CNSA)^57^ of China National GeneBank DataBase (CNGBdb)^58^ with accession number CNP0004258.

## Technical Validation

The completeness of the elephant genomes was evaluated by the BUSCO^59^ (v5.2.2) analysis with mammalia_odb10 data set, scoring at 95.1 ± 1.1% (Table 5). The Merqury^60^ (release 20200430) k-mer analysis and PacBio long reads’ alignments (genome regions with PacBio long-read coverage over 10× were considered as accurate assembled regions^61^) were used for evaluating the genome assembly accuracy of this genome (Table 5, Supplementary Table 9). The completeness of the genome and gene set was also evaluated using the database of mammalia_odb10 through BUSCO. The two chromosome-level genomes scored 96.3% and 95.2%, respectively (Supplementary Table 10). The NUCmer program from the MUMmer^32^ (v4.0.0rc1) was performed for Syntenic blocks screening, and these identified syntenic blocks were filtered by using the delta-filter program from the MUMmer^32^ (v4.0.0rc1) with parameters “-i 90 -l 5000”, to assist in demonstrating the haplotype effect (Supplementary Fig. 4).

**Table 5:** Summary of genome quality assessments.

Genome	BUSCO scores	Completeness	Long reads mapping rate
EmaxH1	94.0%	92.79%	99.46
EmaxH2	95.8%	97.07%	98.69
EmaxG	95.9%	97.17%	/
LafrH1	93.9%	92.77%	99.52
LafrH2	96.1%	96.60%	99.00
LafrG	96.2%	96.83%	/

### Supplementary information

Supplementary information

## Data Availability

No specific script was used in this work. The codes and pipelines used in data processing were all executed according to the manual and protocols of the corresponding bioinformatics software. The specific versions of software have been described in Methods.
