# Supplementary material for: Haplotype-resolved chromosome-scale genomes of the Asian and African Savannah Elephants
Source: Sci Data. 2024 Jan 11;11:63. doi: 10.1038/s41597-023-02729-4 (PMC10784532; doi:10.1038/s41597-023-02729-4)

Supplementary information
Haplotype-resolved chromosome-scale genomes of the Asian and African Savannah Elephants

**Supplementary Table 1.** Information about the RNASeq data used to annotate expressed genes.	Page 2
**Supplementary Table 2.** Sequence lengths of the pseudochromosomes of *E. maximus* chromosome-scale (EmaxG) and haplotype-resolved (EmaxH1 & EmaxH2) genomes.	Page 2
**Supplementary Table 3.** Sequence lengths of the pseudochromosomes of *L. africana* chromosome-scale (LafrG) and haplotype-resolved (LafrH1 & LafrH2) genomes.	Page 3
**Supplementary Table 4.** Genomic statistics of *E. maximus* (EmaxG) and *L. Africana* (LafrG) chromosome-scale genomes.	Page 4
**Supplementary Table 5.** Statistics of repeats in the *E. maximus* and *L. africana* chromosome-scale genomes (EmaxG and LafrG).	Page 5
**Supplementary Table 6.** Transposable elements in the *E. maximus* and *L. Africana* chromosome-scale genomes (EmaxG and LafrG).	Page 5
**Supplementary Table 7.** Functional annotation of gene sets for the *E. maximus* and *L. africana* (EmaxG and LafrG).	Page 5
**Supplementary Table 8.** Statistics of ncRNA annotation for the *E. maximus* and *L. africana* (EmaxG and LafrG).	Page 6
**Supplementary Table 9.** Base quality and completeness statistics for *E. maximus* (EmaxG, EmaxH1 & EmaxH2) and *L. africana* (LafrG, LafrH1 & LafrH2) genome assemblies estimated by the Merqury software.	Page 6
**Supplementary Table 10.** BUSCO analysis of the *E. maximus* and *L. africana* gene sets (EmaxG and LafrG).	Page 6
**Supplementary Fig. 1.** The K-mer spectra (k-mer=17) of *E. maximus* and *L. africana* genome. The final estimated genome size is 3.44 Gb for E. maximus and 3.50 Gb for L. africana.	Page 7
**Supplementary Fig. 2.** Chromosomal sequencing depth of three genomes of *E. maximus* and *L. africana* (aligned to DNBSEQ reads).	Page 7
**Supplementary Fig. 3.** Gene families among the 16 species.	Page 8
**Supplementary Fig. 4.** Dot plot between two sets of haplotype genomes of *E. maximus* and *L. africana* (plotted by pafCoordsDotPlotly).	Page 8

Supplementary Table 1. Information about the RNASeq data used to annotate expressed genes.

Species	Tissue type	BioSample of NCBI	Accession of GSA	Sample accession of CNGBdb
Elephas maximus	Blood	SAMN37042392	SAMC2999257	CNS0818843
Loxodonta africana	Muscle	SAMN37042393	SAMC2999258	CNS0818844

Supplementary Table 2. Sequence lengths of the pseudochromosomes of *E. maximus* chromosome-scale (EmaxG) and haplotype-resolved (EmaxH1 & EmaxH2) genomes.

EmaxG	Length	EmaxH1	Length	EmaxH2	Length	Note
HiC_scaffold_1	244,444,223	HiC_scaffold_1	241,337,252	HiC_scaffold_1	240,666,130	
HiC_scaffold_2	236,900,082	HiC_scaffold_2	234,859,177	HiC_scaffold_2	236,713,967	
HiC_scaffold_3	216,475,380	HiC_scaffold_3	215,391,841	HiC_scaffold_3	215,897,700	
HiC_scaffold_4	192,023,614	HiC_scaffold_4	192,150,293	HiC_scaffold_4	192,429,122	
HiC_scaffold_5	176,912,950	/	/	HiC_scaffold_5	176,905,053	X
HiC_scaffold_6	164,582,382	HiC_scaffold_5	163,508,910	HiC_scaffold_6	165,475,714	
HiC_scaffold_7	141,151,174	HiC_scaffold_6	141,065,805	HiC_scaffold_7	141,171,883	
HiC_scaffold_8	135,247,377	HiC_scaffold_7	133,565,386	HiC_scaffold_8	133,625,077	
HiC_scaffold_9	133,183,069	HiC_scaffold_8	132,062,635	HiC_scaffold_9	131,798,559	
HiC_scaffold_10	130,469,234	HiC_scaffold_9	124,428,925	HiC_scaffold_10	121,677,346	
HiC_scaffold_11	117,232,897	HiC_scaffold_10	115,751,594	HiC_scaffold_11	115,916,506	
HiC_scaffold_12	110,248,437	HiC_scaffold_11	109,584,020	HiC_scaffold_12	110,064,207	
HiC_scaffold_13	107,301,976	HiC_scaffold_12	105,255,511	HiC_scaffold_13	105,442,146	
HiC_scaffold_14	95,698,955	HiC_scaffold_15	93,101,884	HiC_scaffold_15	95,070,156	
HiC_scaffold_15	94,434,016	HiC_scaffold_14	94,339,705	HiC_scaffold_16	94,292,862	
HiC_scaffold_16	89,755,565	HiC_scaffold_13	101,723,068	HiC_scaffold_14	100,362,479	
HiC_scaffold_17	87,937,754	HiC_scaffold_16	88,310,170	HiC_scaffold_17	87,930,897	
HiC_scaffold_18	85,798,791	HiC_scaffold_17	85,723,928	HiC_scaffold_18	85,767,341	
HiC_scaffold_19	83,706,521	HiC_scaffold_18	80,948,795	HiC_scaffold_19	82,425,179	
HiC_scaffold_20	81,371,816	HiC_scaffold_19	80,622,026	HiC_scaffold_21	79,431,878	
HiC_scaffold_21	81,117,973	HiC_scaffold_23	74,840,390	HiC_scaffold_24	74,985,184	
HiC_scaffold_22	80,514,477	HiC_scaffold_20	79,827,973	HiC_scaffold_20	80,278,519	
HiC_scaffold_23	80,119,244	HiC_scaffold_21	79,170,510	HiC_scaffold_22	79,251,652	
HiC_scaffold_24	79,328,989	HiC_scaffold_22	77,704,157	HiC_scaffold_23	77,536,291	
HiC_scaffold_25	65,879,712	HiC_scaffold_24	65,525,319	HiC_scaffold_25	65,846,836	
HiC_scaffold_26	64,594,098	HiC_scaffold_25	64,906,329	HiC_scaffold_26	64,409,056	
HiC_scaffold_27	49,280,587	HiC_scaffold_26	47,464,954	HiC_scaffold_27	48,441,618	
HiC_scaffold_28	39,050,260	HiC_scaffold_27	41,306,124	HiC_scaffold_28	40,475,180	
HiC_scaffold_29	10,914,511	HiC_scaffold_28	10,383,462	/	/	Y

Supplementary Table 3. Sequence lengths of the pseudochromosomes of *L. africana* chromosome-scale (LafrG) and haplotype-resolved (LafrH1 & LafrH2) genomes.

LafrG	Length	LafrH1	Length	LafrH2	Length	Note
HiC_scaffold_1	238,728,000	HiC_scaffold_1	238,717,381	HiC_scaffold_1	238,749,611	
HiC_scaffold_2	232,794,839	HiC_scaffold_2	231,079,901	HiC_scaffold_2	232,198,865	
HiC_scaffold_3	210,954,170	HiC_scaffold_3	210,295,606	HiC_scaffold_3	208,962,500	
HiC_scaffold_4	191,867,476	HiC_scaffold_4	191,602,599	HiC_scaffold_4	191,751,135	
HiC_scaffold_5	174,566,310	/	/	HiC_scaffold_5	174,102,010	X
HiC_scaffold_6	162,967,661	HiC_scaffold_5	162,904,881	HiC_scaffold_6	162,775,699	
HiC_scaffold_7	140,574,937	HiC_scaffold_6	140,888,172	HiC_scaffold_7	140,828,693	
HiC_scaffold_8	136,236,017	HiC_scaffold_7	134,179,964	HiC_scaffold_8	134,988,986	
HiC_scaffold_9	132,885,000	HiC_scaffold_8	132,863,934	HiC_scaffold_9	132,930,693	
HiC_scaffold_10	122,446,792	HiC_scaffold_9	122,926,248	HiC_scaffold_10	122,288,900	
HiC_scaffold_11	113,355,870	HiC_scaffold_10	112,874,280	HiC_scaffold_11	112,645,645	
HiC_scaffold_12	108,717,402	HiC_scaffold_11	108,532,692	HiC_scaffold_12	109,954,823	
HiC_scaffold_13	105,220,632	HiC_scaffold_12	105,555,763	HiC_scaffold_13	105,293,133	
HiC_scaffold_14	94,262,000	HiC_scaffold_13	92,637,380	HiC_scaffold_14	92,502,645	
HiC_scaffold_15	92,483,839	HiC_scaffold_14	91,821,857	HiC_scaffold_15	92,047,325	
HiC_scaffold_16	87,907,454	HiC_scaffold_15	86,836,884	HiC_scaffold_16	87,956,823	
HiC_scaffold_17	84,954,068	HiC_scaffold_16	85,329,339	HiC_scaffold_17	85,153,000	
HiC_scaffold_18	82,402,776	HiC_scaffold_17	82,295,820	HiC_scaffold_18	82,147,193	
HiC_scaffold_19	81,292,815	HiC_scaffold_18	81,266,816	HiC_scaffold_19	81,321,092	
HiC_scaffold_20	80,265,500	HiC_scaffold_19	79,514,223	HiC_scaffold_20	80,116,497	
HiC_scaffold_21	79,060,500	HiC_scaffold_20	79,020,500	HiC_scaffold_21	79,007,269	
HiC_scaffold_22	78,462,839	HiC_scaffold_21	78,761,680	HiC_scaffold_22	78,889,000	
HiC_scaffold_23	78,454,193	HiC_scaffold_22	77,525,720	HiC_scaffold_23	78,179,989	
HiC_scaffold_24	76,018,500	HiC_scaffold_23	75,969,500	HiC_scaffold_24	75,897,022	
HiC_scaffold_25	64,538,454	HiC_scaffold_24	64,570,500	HiC_scaffold_25	64,441,000	
HiC_scaffold_26	64,048,956	HiC_scaffold_25	64,024,409	HiC_scaffold_26	63,975,446	
HiC_scaffold_27	47,586,831	HiC_scaffold_26	47,645,997	HiC_scaffold_27	46,899,954	
HiC_scaffold_28	32,304,669	HiC_scaffold_27	32,767,140	HiC_scaffold_28	32,632,188	
HiC_scaffold_29	8,551,807	HiC_scaffold_28	9,715,282	/	/	Y

Supplementary Table 4. Genomic statistics of *E. maximus* (EmaxG) and *L. Africana* (LafrG) chromosome-scale genomes.

Genome	Item	Scaffold length(bp)	Number	Contig length(bp)	Number	Ngenome length(bp)	Number
EmaxG	max_len	244,444,223	/	236,900,082	/	/	/
	N10	236,900,082	2	155,239,616	2	236,900,082	2
	N20	216,475,380	3	121,290,821	5	216,475,380	3
	N30	176,912,950	5	96,690,608	8	176,912,950	5
	N40	141,151,174	7	84,365,969	11	141,151,174	7
	N50	130,469,234	10	77,194,844	16	127,268,234	10
	N60	107,301,976	13	61,219,888	20	107,301,976	13
	N70	89,755,565	16	48,976,704	27	89,755,565	16
	N80	81,371,816	20	33,078,161	35	81,371,816	20
	N90	79,328,989	24	14,785,507	49	79,328,989	24
	Total_length	3,377,773,971	/	3,377,713,718	/	/	/
	Number>=100bp	45	/	176	/	/	/
	Number>=2000bp	45	/	176	/	/	/
	GC_rate	0.411	/	0.411	/	/	/
	Estimated Genomesize	3,442,174,663	/	/	/	/	/
LafrG	max_len	238,728,000	/	232,641,661	/	/	/
	N10	232,794,839	2	199,318,026	2	232,794,839	2
	N20	210,954,170	3	132,772,000	4	210,954,170	3
	N30	174,566,310	5	111,759,839	7	174,566,310	5
	N40	140,574,937	7	86,591,870	10	140,574,937	7
	N50	122,446,792	10	71,750,044	14	122,446,792	10
	N60	105,220,632	13	59,085,839	19	105,220,632	13
	N70	87,907,454	16	44,039,439	26	87,907,454	16
	N80	80,265,500	20	35,048,000	34	80,265,500	20
	N90	76,018,500	24	13,880,000	49	76,018,500	24
	Total_length	3,314,059,562	/	3,313,776,217	/	/	/
	Number>=100bp	743	/	1,324	/	/	/
	Number>=2000bp	695	/	1,267	/	/	/
	GC_rate	0.410	/	0.410	/	/	/
	Estimated Genomesize	3,498,857,250	/	/	/	/	/

Supplementary Table 5. Statistics of repeats in the *E. maximus* and *L. africana* chromosome-scale genomes (EmaxG and LafrG).

Type	EmaxG		LafrG	
	Length (bp)	% of genome	Length (bp)	% of genome
Trf	140,228,744	4.15	79,715,005	2.41
Repeatmasker	1,436,808,907	42.54	1,420,543,224	42.86
Proteinmask	597,777,156	17.70	602,415,858	18.18
Denovo	1,713,856,377	50.74	1,599,252,171	48.26
Total	2,458,799,323	72.79	2,340,927,131	70.64

Supplementary Table 6. Transposable elements in the *E. maximus* and *L. africana* chromosome-scale genomes (EmaxG and LafrG).

Type		Repbase Tes		TE proteins		*De novo*		Combined Tes	
		Length (bp)	% in genome	Length (bp)	% in genome	Length (bp)	% in genome	Length (bp)	% in genome
EmaxG	DNA	63,673,498	1.89	4,208,969	0.12	57,596,407	1.71	98,864,930	2.93
	LINE	1,097,145,508	32.48	574,732,289	17.02	1,160,844,187	34.37	1,818,056,280	53.82
	SINE	134,422,181	3.98	-	-	145,537,485	4.31	220,195,495	6.52
	LTR	168,992,577	5.00	25,571,578	0.76	459,424,604	13.60	502,367,633	14.87
	Other	119	0.00	-	-	-	-	119	0.00
	Unknown	-	-	-	-	37,348,324	1.11	37,348,324	1.11
	Total	1,436,808,907	42.54	597,777,156	17.70	1,711,579,755	50.67	2,375,283,282	70.32
LafrG	DNA	50,436,817	1.52	4,291,527	0.13	24,166,179	0.73	62,963,140	1.90
	LINE	1,081,730,758	32.64	578,450,622	17.45	1,027,567,281	31.01	1,806,545,100	54.51
	SINE	133,540,433	4.03	0	0.00	20,853,218	0.63	147,446,993	4.45
	LTR	169,292,687	5.11	25,927,257	0.78	689,405,092	20.80	735,879,514	22.20
	Other	115	0.00	0	0.00	0	0.00	115	0.00
	Unknown	0	0.00	0	0.00	21,973,580	0.66	21,973,580	0.66
	Total	1,420,543,224	42.86	602,415,858	18.18	1,597,744,581	48.21	2,291,962,134	69.16

Supplementary Table 7. Functional annotation of gene sets for the *E. maximus* and *L. africana* (EmaxG and LafrG).

Genome	Values	Total genes	Swissprot-Annotated	KEGG-Annotated	TrEMBL-Annotated	Interpro-Annotated	GO-Annotated	Overall annotated
**EmaxG**	**Number**	22,177	21,648	19,839	21,900	21,993	16,753	22,135
	**Percentage**	100%	97.61%	89.46%	98.75%	99.17%	75.54%	99.81%
**LafrG**	**Number**	22,142	21,621	19,825	21,859	21,961	16,715	22,100
	**Percentage**	100%	97.65%	89.54%	98.72%	99.18%	75.49%	99.81%

Supplementary Table 8. Statistics of ncRNA annotation for the *E. maximus* and *L. africana* (EmaxG and LafrG).

Type		EmaxG				LafrG			
		Number	Average length (bp)	Total length (bp)	% of genome	Number	Average length (bp)	Total length (bp)	% of genome
miRNA		2467	78.2541548	193053	0.005420	2436	78.3435961	190845	0.005351
tRNA		56204	74.5344637	4189135	0.117604	56158	74.5576587	4187009	0.11739
rRNA	rRNA	1026	367.568226	377125	0.010587	1441	396.013879	570656	0.015999
	18S	124	941.048387	116690	0.003276	149	1107.98658	165090	0.004629
	28S	674	347.750742	234384	0.00658	1000	370.811	370811	0.010396
	5.8S	58	156.034483	9050	0.000254	87	156.057471	13577	0.000381
	5S	170	100.005882	17001	0.000477	205	103.307317	21178	0.000594
snRNA	snRNA	2432	98.1254112	238641	0.006699	1859	102.870898	191237	0.005362
	CD-box	1336	80.7956587	107943	0.003030	773	81.5976714	63075	0.001768
	HACA-box	157	138.203822	21698	0.000609	159	137.861635	21920	0.000615
	splicing	911	115.095499	104852	0.002944	899	113.562848	102093	0.002862

Supplementary Table 9. Base quality and completeness statistics for *E. maximus* (EmaxG, EmaxH1 & EmaxH2) and *L. africana* (LafrG, LafrH1 & LafrH2) genome assemblies estimated by the Merqury software.

Genome	K-mers uniquely found only in the assembly	K-mers found in both assembly and the read set	QV	Error rate	Solid k-mers in the assembly	Total solid k-mers in the read set	Completeness (%)
EmaxH1	790,798	3,125,712,465	49.1905	1.2049e-05	2,126,053,450	2,291,294,436	92.7883
EmaxH2	825,563	3,335,671,432	49.286	1.17869e-05	2,224,163,565	2,291,294,436	97.0702
EmaxG	901,229	3,377,710,438	48.9595	1.27072e-05	2,225,672,852	2,291,294,436	97.136
LafrH1	811,449	3,103,973,771	49.0482	1.24502e-05	2,130,453,045	2,296,394,531	92.7738
LafrH2	859,343	3,267,284,874	49.0219	1.25261e-05	2,218,223,033	2,296,394,531	96.5959
LafrG	983,806	3,313,749,737	48.4957	1.41394e-05	2,223,637,259	2,296,394,531	96.8317

Supplementary Table 10. BUSCO analysis of the *E. maximus* and *L. africana* gene sets (EmaxG and LafrG).

Genome	BUSCO scores	dataset
EmaxG	C:96.3%[S:95.7%,D:0.6%],F:1.7%,M:2.0%,n:9226	mammalia_odb10
LafrG	C:95.2%[S:94.6%,D:0.6%],F:2.3%,M:2.5%,n:9226	mammalia_odb10

Supplementary Fig. 1. The K-mer spectra (k-mer=17) of *E. maximus* and *L. africana* genome. The final estimated genome size is 3.44 Gb for *E. maximus* and 3.50 Gb for *L. africana*.

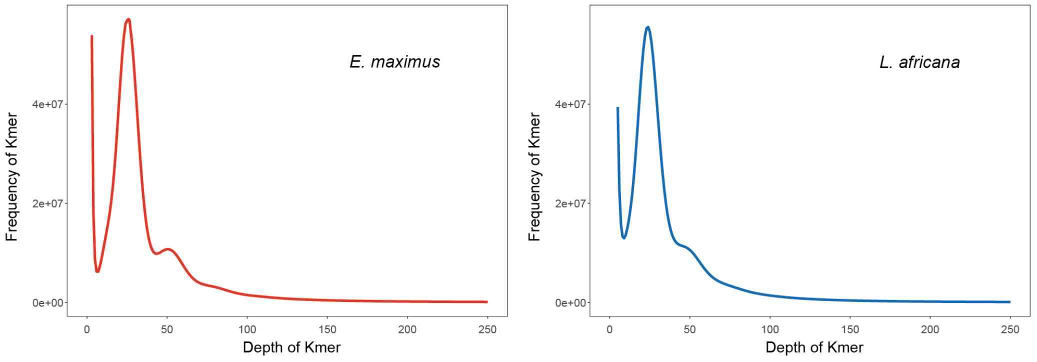

Supplementary Fig. 2. Chromosomal sequencing depth of three genomes of *E. maximus* and *L. africana* (aligned to DNBSEQ reads).

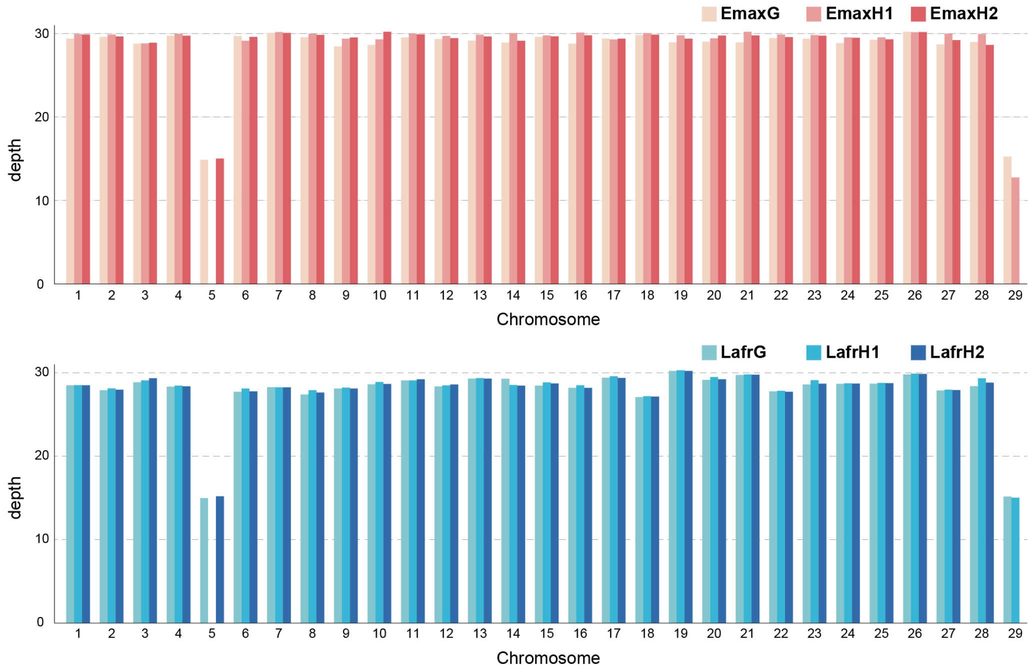

Supplementary Fig. 3. Gene families among the 16 species.

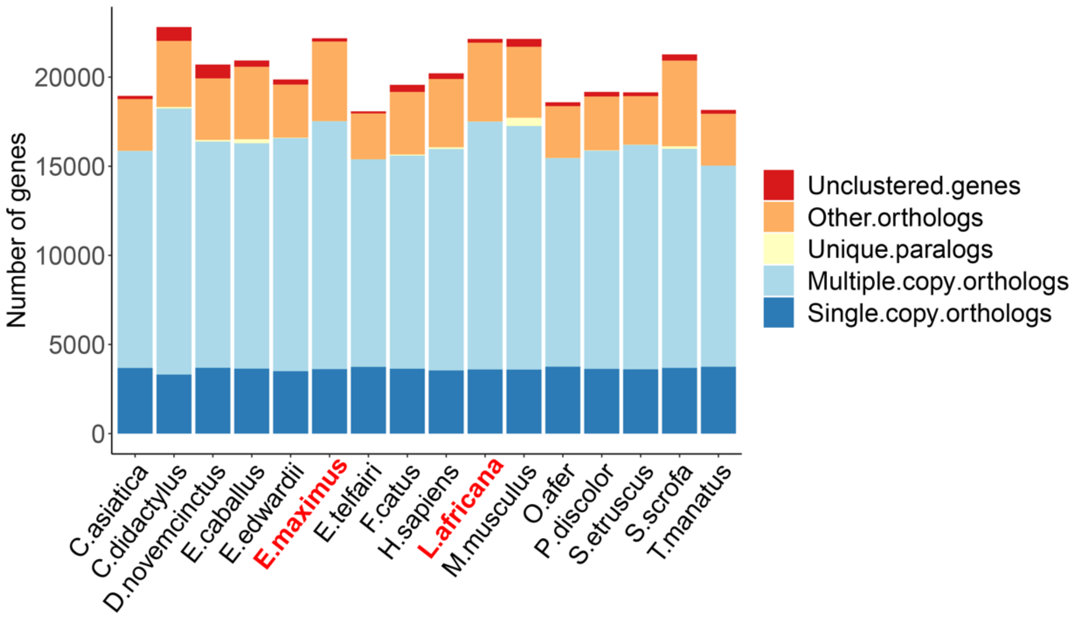

Supplementary Fig. 4. Dot plot between two sets of haplotype genomes of *E. maximus* and *L. africana* (plotted by pafCoordsDotPlotly).

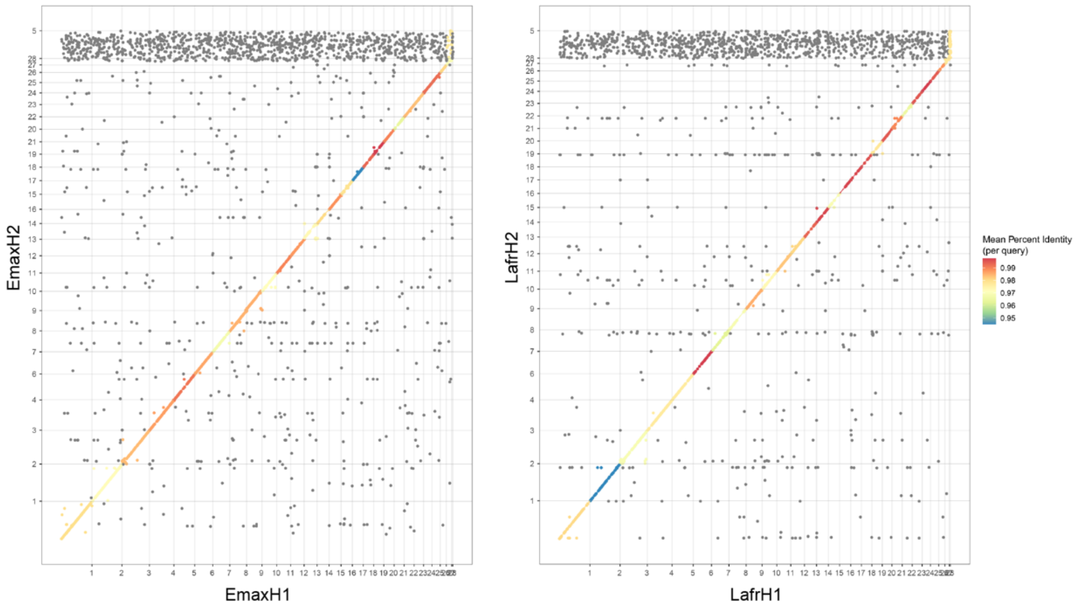

Supplement: Supplementary file 1 — Supplementary information [file 41597_2023_2729_MOESM1_ESM.pdf]
